# Inequalities in health care utilization for common childhood illnesses in Ethiopia: evidence from the 2011 Ethiopian Demographic and Health Survey

**DOI:** 10.1186/s12939-017-0561-7

**Published:** 2017-04-21

**Authors:** Asmamaw Atnafu Ayalneh, Dagnachew Muluye Fetene, Tae Jin Lee

**Affiliations:** 1 Department of Public Health Science, Graduate School of Public Health, Seoul National University, Seoul, South Korea; 2 College of Medicine and Health Sciences, University of Gondar, Gondar, Ethiopia; 3 School of Public Health, University of Queensland, Brisbane, Australia; 4 Institute of Health and Environment, Seoul National University, Seoul, South Korea

**Keywords:** Inequalities, Common childhood illnesses, Health service utilization, Ethiopia

## Abstract

**Background:**

Globally, 5.9 million children under the age of five died in 2015. More than half and almost one-third of those deaths occurred in sub-Saharan Africa and South Asia, respectively. Diarrhea and Pneumonia, which were the major causes of the problem, accounted for more than two million deaths of the world’s youngest children every year. Like other developing countries, child health services utilization is low in Ethiopia. The aim of this study was to identify the determinant factors for the inequalities in medical treatment seeking behavior for common childhood illnesses in Ethiopia.

**Methods:**

Data were obtained from the Ethiopian Demographic and Health Survey (EDHS) 2011. All children who had diarrhea, cough, and fever in the 2 weeks preceding the survey were included. A total of 1620 children with diarrhea, 2082 with fever, and 2134 with cough were included in the analyses. Multivariate logistic regression with a 95% confidence interval, adjusted odds-ratio, and a *P* < 0.05 were used to determine the independent effect of each variable.

**Results:**

Household wealth-status, maternal and paternal education, and religion were found to be associated with the inequality in the use of child health services. Respondents from households with the richest, richer, and middle wealth status had higher odds of seeking medical treatment for childhood diarrhea, cough, and fever than that of the poorest ones. Maternal and paternal educational status was also associated with medical treatment seeking behavior for childhood diarrhea and fever, respectively.

**Conclusion:**

Household wealth and educational status of parents were possible determinant factors for the inequalities observed in health care seeking behavior. Policy interventions aimed at improving the appropriate medical treatment seeking behavior for common childhood illnesses are desirable. Practical economic policies aimed at moving those in the lower wealth quintile are essential to bridge the gap between the rich and the poor. Studies comprising qualitative and quantitative methods are recommended to further explore other determinants of health care utilization.

**Electronic supplementary material:**

The online version of this article (doi:10.1186/s12939-017-0561-7) contains supplementary material, which is available to authorized users.

## Background

Globally, 5.9 million children under the age of five died in 2015. Almost 99% of these deaths occurred in developing countries. More than half and almost one-third of them occurred in sub-Saharan Africa and South Asia, respectively [1]. Despite the global reduction in the under-five mortality since 1990–2013, 223 million children died from pneumonia, preterm birth complications, birth asphyxia, diarrhea and malaria before celebrating their fifth birthday [2, 3]. Diarrhea and Pneumonia are the major killers of the world’s youngest children [4, 5]. Burkina Faso, the Democratic Republic of Congo (DRC), Ethiopia, Nigeria, Tanzania, and Uganda together accounted for almost 53% of the world’s childhood under-five mortality [6]. Pneumonia and diarrhea have long been considered as the major killers of young children. However, these deaths could be largely prevented through an optimal breastfeeding practice, adequate nutrition, vaccination, proper personal hygiene, safe drinking water, and basic sanitation. Furthermore, once the child gets sick, death can be prevented through cost effective and life-saving medical treatments [4].

Ethiopia as a developing country has experienced high economic growth over the last few decades but remains a poor country with a high burden of diseases [7]. The majority of the inhabitants are rural dweller. According to a 2007 census, the main religions in Ethiopia are Christian (62.8%); 43.5% Ethiopian Orthodox, 19.3% other, followed by Islam (33.9%), Traditional (2.6%) and other (0.6%). The country has a three-tier delivery system for their health care delivery: level one (district health system), level-two (general hospitals) and level three (specialized hospitals). Each of the health care systems is connected by a referral system [8]. The Ethiopian government is the major manager of health resources, and government health facilities are the major recipients of health spending. Moreover, curative services are the major targets of health expenditure (51.6%) [9]. Resources towards the health care systems are highly dependent on donations from the rest of the world and household expenditure. The 5^Th^ (2010/11) National Health Account (NHA) report showed that foreign donation accounts for almost 50% of the financing source for general health care, followed by households (36%), and the domestic government (15.6%).

Despite all this challenges, Ethiopia has improved the health of its children and achieved target 4 of the Millennium Development Goal (under-five mortality rate) by reducing the under- five mortality from the 1990 estimate of 204 deaths/1000 live births by 67%. According to the UN Inter-agency Group’s 2013 mortality estimate report, Ethiopia’s under-five, infant, and neonatal mortality rates were 68, 44, and 28 per 1000 live births, respectively [10]. Notwithstanding current improvements, approximately half a million children under-5 years of age die every year in Ethiopia, 120,000 of whom die in the first month of their life from preventable diseases due to poor access to the health system [2]. Acute respiratory infection and diarrhea are among the major causes of the under-five mortality in Ethiopia. Despite Ethiopia’s achievement in the reduction of child mortality, essential interventions such as case management of acute respiratory infection (ARI) and diarrhea are still low [10]. In the 2011 EDHS report, the general out-patient health care utilization per year was only 0.3 visits. Moreover, only 27% of the children under the age of five with symptoms of ARI sought counsel from a health care facility or provider. Similarly, one out of four febrile children and 32% of children with diarrhea sought care from a health care facility or provider [10, 11].

Numerous studies have investigated the main determinants of child health service utilization in developing countries. Higher household economic status, better maternal education, and intended pregnancy have a positive association with child health service utilization [12–17]. Advanced maternal age, child age, and rural residence are also mentioned as determinant factors for child health care service utilization [14, 16, 18].

The health care seeking behavior of a community for the management of childhood infectious diseases is highly influenced by a community’s socio-economic position and economic development [5]. Maternal or caregiver’s knowledge has an influence on the early management and treatment of fever [19, 20]. According to a research finding from Ethiopia, socio-economic status and religious beliefs were found to be the determinants of health care seeking behavior for childhood illnesses [21]. An additional study in Ethiopia documented that the main reasons for not seeking medical treatment from health care facilities as reported by mothers/caregivers were considering illnesses as not serious, lack of money, and negative previous experiences from using health care facilities [22].

Child death from preventable causes, such as diarrhea, fever, and cough, still remains high. As a result, health care seeking behavior, health service utilization, and their determinants in the community need to be explored. The aim of this study was, therefore, to identify the main determinant factors that influence equitable medical treatment seeking behavior for common childhood illnesses in Ethiopia using nationally representative data.

## Methods

### Data description and sampling procedure

Data were obtained from the 2011 Ethiopian Demographic and Health Survey (EDHS). EDHS is part of the worldwide MEASURE DHS project funded by the United States Agency for International Development (USAID). It was conducted by the agencies of the Federal Ministry of Health and the Central Statistical Agency (CSA) of Ethiopia from September 2010 to June 2011 by interviewing a nationally representative sample of 16,515 women from 17,702 households [11]. The Demographic and Health Survey (DHS) data of children under-five were used for the statistical analysis. A total of 1620 children with diarrhea, 2082 with cough, and 2134 with fever were included in the final analysis.

The DHS used a population-based, cross-sectional data collection method. The sample for the 2011 EDHS was designed to provide population and health indicators at national and regional levels. The sample was representative of 11 geographic/administrative regions; the samples were selected using a stratified, two-stage cluster design, and enumeration areas (EAs) were the sampling units for the first stage. The samples included 624 enumerator areas (EAs): 187 urban and 437 rural. Households comprised the second stage of the sampling. A complete list of households was drawn up for each of the 624 selected EAs, and representative samples of the 17,817 households were selected. All women aged 15–49 years and all men aged 15–59 years who were either permanent residents of the selected households or visitors who stayed in the households the night before the survey were eligible for the interview. With the parent’s or guardian’s consent, children aged 0 to 59 months were made part of the study [11]. A structured and pretested questionnaire (http://dhsprogram.com/pubs/pdf/FR255/FR255.pdf) was used for the data collection. Interviews were conducted in the local language [11].

### Variable definition

#### Dependent variables

Medical treatments sought for childhood diarrhea, fever, and cough were the three main outcome variables of interest in this study. A child with diarrhea, fever, and cough was identified in the DHS data through respondents/mothers reporting whether the child had diarrhea and/or fever, and/or cough in the 2 weeks preceding the survey period. Medical treatment seeking behaviors for those childhood illnesses were assessed by interviewing respondents/mothers. The respondents/mothers were asked about whether they had sought medical treatment when the child was sick with the above-mentioned childhood illnesses.

#### Independent variables

The predisposing variables were age and sex of the child, maternal and paternal educational status, age of the mother, household religion, household size and number of under-five children. Household wealth and residence were used as enabling factors. The EDHS survey had no household income data; however, household wealth computed from household assets and household characteristics were available. Principal component analysis (PCA) was used to compute wealth index from the household assets and household characteristics. Some of the variables used to measure the household assets were the availability of radio receivers, television sets, mobile phones, non-mobile phones, refrigerators, agricultural land ownership, livestock ownerships and others. Moreover, household characteristics, such as source of drinking water, source of water for cooking and washing, type of toilet facility, type of cooking fuel and others were used [23]. Based on the wealth, households were split into five categories, as poorest, poorer, middle, richer, and richest. To evaluate the effect of mass media information on pediatric health service utilization, we included household radio receiver and television set possession as categorical variables during our analysis.

### Data analysis

The Stata version 14 [STATA/SE 14, StataCorp LP, 4905 Lakeway Drive, College Station, Texas 77845] software was used to carry out the data analysis. The unit of analysis was a child born within the 5 years prior to the data collection period. The analysis was done on weighted data. The weights were used to adjust for sample design effects and the non-response rate and to generate correct estimates of the standard errors. The bivariate analysis was computed to determine the presence of associations between dependent and independent variables. All variables were included in the multivariate logistics model once they were significantly associated (*P* < 0.05) at the bivariate level to determine the independent effect of each variable. The adjusted odds ratio (AOR) with a 95% confidence interval and a *p*-value < 0.05 were used to determine the presence of an association between dependent and independent variables.

## Results

### Descriptive findings of predictor and outcome variables

Out of the 1620 children with diarrhea, 2134 with cough, and 2082 with fever only, 35% (576), 26% (555), and 29% (604) sought medical treatment for diarrhea, cough, and fever, respectively. Almost half of the respondents for childhood diarrhea, cough, and fever were from the poorest and poorer wealth status households. Sixty-nine percent (1125) of the respondents for children with diarrhea, 68% (1451) of the respondents for children with cough, and 68.4% (1425) of the respondents for children with fever had no formal education. The majority of the respondents were Muslim, accounting for 43% (679), 41.6% (888), and 42% (875) of the children who had diarrhea, cough, and fever, respectively. The majority of the households, that is, 81.9% (1326) of those with childhood diarrhea, 82.4% (1758) of those with childhood cough, and 83.4% (1737) of those with childhood fever, had less than 3 under-five children in the household. Of all the respondents, 36% (579) with diarrhea, 36.5% (778) with cough, and 37% (770) with fever owned radio receivers at the household level. However, only 10% (157), 10.1% (216) and 11% (222) of the respondents with childhood diarrhea, cough, and fever, respectively, had television sets in their household. Most of the respondents (85%) were rural residents. The details of the descriptive results are shown in the supplementary table [Additional file 1: Table S1].

### Multivariate analyses

#### Determinants of medical treatment seeking behavior for childhood diarrhea

In the multivariate logistic regression, household wealth-status, maternal education, paternal education, and the age of the children were significantly associated with the medical treatment seeking behavior of the respondents for childhood diarrheal diseases. Out of the 1620 children with diarrhea, only 35% (576) sought medical treatment for diarrheal diseases.

**Fig. 1:**
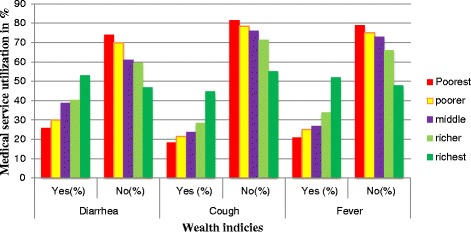
The distribution of wealth indices with corresponding medical care utilization for childhood diarrhea, cough and fever illness

Respondents from households with the richest (AOR = 1.84 (1.11–3.05)), richer (AOR = 1.78 (1.27–2.48)), and middle (AOR = 1.84 (1.33–2.56)) wealth status had higher odds of seeking medical treatment for children with diarrhea than those from households with the poorest wealth status (Fig. 1). Mothers with secondary and higher education had 2.05 and 4.6 times (AOR = 2.05 (1.02–4.12)), (AOR = 4.6, (1.3–9.3)) higher odds of seeking medical treatment than mother’s without education. In addition, paternal higher education had 1.5 times higher odds of seeking medical treatment than the uneducated ones (AOR = 1.5, (0.47–2.22)). Mothers had two times higher odds of seeking medical treatment for children in the 6 to 11 (AOR = 2.09 (1.27–3.43)), 12 to 23 (AOR = 2.23 (1.4–3.55)), 24 to 35 (AOR = 2.04 (1.26–3.32)), and 36–47 (AOR = 2.37 (1.43–3.93)) month age group than for children aged less than 6 months old. Maternal age was negatively associated with the medical treatment seeking behavior for childhood diarrheal disease; mothers aged 35–39 years had a 48% lower odds ratio (AOR = 0.52 (0.27–1.02)) for seeking medical treatment than mothers aged 15–24 years. Moreover, the number of under-five children in the household and the female workload were negatively correlated with medical treatment seeking behavior; however, the association was not statistically significant [Table 1].

**Table 1:** Adjusted logistic regression result on the association of predictor and outcome variables

Predictor variable	OR(95%CI)		
	Diarrhea treatment	Cough treatment	Fever treatment
Household wealth index			
Poorest	1	1	1
Poorer	1.24 [0.88–1.73]	1.27 [0.91–1.76]	1.25 [0.92–1.71]
Middle	1.84 [1.33–2.56]**	1.45 [1.06–2.0]*	1.47 [1.07–2.01]*
Richer	1.78 [1.27–2.48]**	1.78 [1.28–2.47]**	1.83 [1.33–2.52]**
Richest	1.84 [1.11–3.05]*	2.46 [1.56–3.89]**	2.46 [1.57–3.86]**
Maternal highest education			
No education	1	1	1
Primary education	1.13 [0.87–1.47]	1.08 [0.84–1.40]	1.02 [0.79–1.32]
Secondary education	2.05 [1.02–4.12]*	1.03 [0.56–1.87]	1.14 [0.62–2.1]
Higher education	4.60 [1.29–9.03]*	1.59 [0.63–4.01]	2.35 [0.8–6.87]
Husband’s highest education			
No education	1	1	1
Primary education	1.05 [0.82–1.33]	1.06 [0.83–1.34]	1.16 [0.92–1.47]
Secondary education	1.26 [0.78–2.04]	1.35 [0.89–2.07]	1.82 [1.19–2.78]*
Higher education	1.5 [0.47–2.22]*	1.31 [0.68–2.53]	1.34 [0.71–2.55]
Household religion			
Orthodox		1	1
Protestant		1.87 [1.39–2.51]**	1.78 [1.32–2.38]**
Muslim		1.44 [1.13–1.84]**	1.48 [1.15–1.90]**
Other		1.33 [0.48–3.71]	1.06 [0.43–2.6]
Maternal age			
15–19	1	1	1
20–24	0.99 [0.58–1.69]	0.69 [0.42–1.12]	0.95 [0.59–1.51]
25–29	1.09 [0.62–1.92]	0.66 [0.4–1.11]	1.00 [0.61–1.65]
30–34	1.21 [0.65–2.25]	0.85 [0.47–1.51]	1.04 [0.6–1.82]
35–39	0.52 [0.27–1.02]	0.47 [0.25–0.88]*	0.76 [0.42–1.38]
40–44	0.89 [0.42–1.88]	0.75 [0.37–1.52]	1.32 [0.68–2.55]
45–49	0.39 [0.12–1.25]	0.54 [0.2–1.48]	0.74 [0.3–1.8]
Number of household			
Less than 6	1	1	1
6 and above	1.09 [0.84–1.43]	0.81 [0.63–1.04]	0.87 [0.69–1.11]
Number of under-5 member’s			
Less than 3	1		
3 & above	0.87 [0.64–1.18]		
Child birth-order			
First	1	1	1
Second	0.85 [0.58–1.24]	0.92 [0.65–1.28]	0.69 [0.49–0.98]*
Third	0.73 [0.47–1.12]	1.21 [0.82–1.78]	0.72 [0.49–1.06]
Fourth & above	0.80 [0.52–1.22]	0.93 [0.63–1.39]	0.68 [0.46–1.01]
Wanted last child			
Wanted no more	1	1	1
Wanted then	1.40 [0.95–2.07]	1.33 [0.9–1.98]	1.40 [0.96–2.03]
Wanted later	1.25 [0.8–1.96]	1.13 [0.73–1.76]	1.28 [0.84–1.96]
Child-age			
< 6 month	1	1	1
6 month–11 month	2.09 [1.27*–*3.43]**	1.83 [1.2–2.79]*	1.72 [1.1–2.69]*
12 month–23 month	2.23 [1.40–3.55]**	1.52 [1.02–2.28]*	1.73 [1.14–2.61]*
24 month–35 month	2.04 [1.26–3.32]**	1.48 [0.99–2.23]	1.90 [1.24–2.91]**
36 month–47 month	2.37 [1.43–3.93]**	1.20 [0.79–1.92]	1.32 [0.85–2.05]
48 month–59 month	1.57 [0.9–2.77]	1.25 [0.08–1.92]	1.49 [0.95–2.34]
Household has radio			
Yes	1	1	1
No	0.93 [0.73–1.18]	1.02 [0.81–1.29]	1.03 [0.82–1.29]
Household has TV			
No	1	1	1
Yes	0.99 [0.62–1.59]	1.92 [1.30–2.82]**	1.27 [0.85–1.9]
Females workload inside and outside			
Big problem	1		1
Not a big problem	1.24 [0.98–1.58]		1.26 [1.01–1.57]*
Place of residence			
Urban	1	1	1
Rural	0.69 [0.45–1.05]	0.97 [0.65–1.44]	0.80 [0.54–1.17]

*p-value < 0.05

** *p*-value < 0.01

#### Determinants of medical treatment seeking behavior for childhood cough

Out of the 2134 children who had cough, 26% (555) sought medical treatment for the disease. Household wealth status, household religion, maternal age, possession of television sets, and the age of the children showed a statistically significant association with the medical treatment seeking behavior for childhood cough.

Respondents from households with the richest, richer, and middle wealth status had 2.46, 1.78 and 1.45 times higher odds, respectively, of medical treatment seeking behavior than for respondents from households with the poorest wealth status (AOR = 2.46 (1.56–3.89)), AOR = 1.78 (1.28–2.47)), and (AOR = 1.45 (1.06–2.00)) (Fig. 1). Respondents from Protestant (AOR = 1.87 (1.39–2.51)) and Muslim AOR = 1.44 (1.13–1.84)) households had higher odds of medical treatment seeking behavior than respondents from Orthodox Christian households. Older mothers (34–39 years of age) had a 53% lower odds ratio compared to younger mothers (15–24 years of age) (AOR = 0.47 (0.25–0.88)). In addition, children aged 6–11 months (AOR = 1.83 (1.2–2.79)) and 12–23 months (AOR =1.52 (1.02–2.28)) had higher odds of medical treatment seeking behavior than children aged less than 6 months. The presence of television sets in households had almost 2 times higher odds of medical treatment seeking behavior than households without television sets (AOR = 1.92 (1.3–2.82)). Rural residence, larger family households and child birth order were not significantly associated with medical treatment seeking behavior for childhood cough [Table 1].

#### Determinants of medical treatment seeking behavior for childhood fever

Out of the 2082 children with fever, only 29% (604) sought medical treatment. Household wealth status, parental educational status, household religion, child age category, and female workload inside and outside the home were statistically significantly associated with medical treatment seeking behavior for childhood fever.

Respondents from households with the richest, richer, and middle wealth status had 2.46, 1.83, and 1.47 times higher odds of seeking medical treatment, respectively, than respondents from households with the poorest status (AOR = 2.46 (1.57–3.86)), AOR = 1.83 (1.33–2.52)), and (AOR = 1.47 (1.07–2.01)) (Fig. 1). Fathers with secondary education had almost 2 times higher odds of medical treatment seeking behavior than those with no education (AOR = 1.82 (1.19–2.78)). Protestant (AOR = 1.78 (1.32–2.38)) and Muslim (AOR = 1.48 (1.16–1.9)) respondents had higher odds of medical treatment seeking behavior than that of Orthodox Christians. Mothers had a 31% lower odds ratio for seeking medical treatment for second-born children compared to first-born children (AOR = 0.69 (0.49–0.98)). Mothers had almost 2 times higher odds of seeking medical treatment for children aged 24–35 (AOR = 1.9 (1.24–2.91)), 12–23 (AOR = 1.73 (1.14–2.61)) and 6–11 (AOR = 1.72 (1.1–2.69)) months than for children aged less than 6 months. Moreover, females with a low workload inside and outside the home had 1.3 time higher odds of seeking medical treatment than females with a higher workload for childhood fever (AOR = 1.26 (1.01–1.57)). Maternal education, household size, availability of radio receivers and television sets, and urban residence were not significantly associated with medical treatment seeking behavior for childhood fever [Table 1].

## Discussion

This study identified important possible factors that lead to inequalities in medical care utilization for childhood diarrhea, fever, and cough in Ethiopia. In this study, household wealth status, maternal education, paternal education, maternal age, childhood age, childhood birth order, and maternal workload were identified as factors leading to inequality in health care utilization.

Household wealth status was consistently associated with the three outcome variables, in other words, medical treatment seeking behaviors for childhood diarrhea, cough, and fever. Mothers from households with the richest, richer, and middle wealth status had higher odds of seeking medical treatment than the ones from the poorest household. This finding is consistent with studies in Uganda and Ethiopia which showed that delays in care seeking for childhood fever were higher in the lowest socio-economic status than in the highest one [14, 15]. Moreover, this finding is in agreement with other studies in developing and developed countries showing the effect of wealth on medical treatment seeking behavior [4, 5, 13, 24–28]. Household wealth status as a measure of relative economic status has been identified as the most significant predictor of medical treatment for childhood illnesses in several studies [6, 27, 29–31]. The inequality resulting from the wealth status of the households can be explained as follows: wealth as a proxy measure of income has a positive influence on the utilization of health care services, whereas a lack of financial resources can create barriers to accessing services. Even though health care is given at a lower user fee in the rural parts of Ethiopia, additional costs related to transportation and medication challenge a household’s ability to pay; hence, this might prevent the poorest segment of the population from seeking health services.

Maternal and paternal educational status is associated with inequalities in medical treatment seeking behavior for children with diarrhea. This finding is in agreement with findings from several studies in Sub-Saharan Africa, Uganda, Nairobi, and India which identified low parental education as a predisposing factor to low medical care seeking behavior for childhood diarrheal diseases [5, 14, 16, 18, 31, 32]. This finding is also supported by studies showing that low maternal educational level in Ethiopia is correlated with low maternal health service utilization [33–37]. In addition, Ethiopian women with higher education have higher odds of utilizing health facilities than women without education [28]. Education can be assumed to be associated with an increased awareness of illnesses, symptoms, and availability of services. Moreover, educational level is a good proxy of socioeconomic position by enhancing the ability to cope with the various costs involved [38]. In addition, education is likely to enhance female autonomy so that women develop greater confidence and capabilities to make decisions regarding their children’s health [39].

Advanced maternal age was negatively correlated with health care seeking behavior for children with cough. This finding is in agreement with the findings from Nigeria, Rwanda, and Ethiopia that showed that the older the mother is, the lower the health care seeking behavior is [14, 16, 29, 40]. This can be explained as follows: older mothers might prefer using their previous life experiences to treat their children rather than visit health facilities.

In this study, the ages of the children had a significant association with seeking medical treatment for children with diarrhea, cough, and fever. This finding is in agreement with the findings from studies conducted in Uganda and the Philippines that show mothers had lower health care seeking behavior for younger children than for older children [14, 32]. In this study, maternal medical treatment seeking behavior was found to be less likely when the child was younger. This might be due to the fact that frequent illnesses in younger children result in the mothers’ reluctance to seek medical treatment, and thus, they tend to utilize their own traditional solutions [27]. We found that paternal educational status was associated with medical care seeking behavior for children with fever. This finding is consistent with the finding of a study conducted in the Philippines that shows better paternal education leads to higher medical care seeking attempts compared to fathers’ with a lower educational status [32]. The explanation for this is the same as the explanation on how maternal education affects health care utilization which is mentioned above.

Our finding suggests female workload is one of the main factors that deter maternal medical treatment seeking behavior for their children with fever. This can be explained as follows: if mothers or caregivers are very busy inside and outside of the home, they might not have time to take care of their sick children. Household religion was not associated with the health care seeking behavior of mothers for children with diarrhea. However, in the case of children with fever and cough, Protestant and Muslim households had higher odds of seeking medical treatment than Orthodox Christians. This finding is consistent with that of a study that briefly explained the effect of culture and religion on health and illness [37]. The reason could be that Orthodox Christians might prefer visiting traditional healers, such as holy-water found in a church, before visiting modern health care facilities. Thus, this could be an avenue for further research on cultural and religious factors and their effects on the medical care seeking behavior of mothers in Ethiopia. Household possession of televisions was significantly associated with health care utilization for children with cough. This might be related to the fact that accessing information about coughing on television could increase peoples’ awareness about the value of seeking health care services.

### Limitations and strengths of this study

Although this study highlighted key factors influencing health care seeking behavior, it does have some limitations. First, we utilized cross-sectional data that might limit our conclusion on the causal association between predictor and outcome variables. Second, the analysis was based on self-reported childhood morbidity due to diarrhea, fever and cough which could be subject to recall bias. Despite these limitations, a strength of our study, unlike other previous studies in Ethiopia, is that it used the DHS data, which includes nationally representative data, enabling the findings to be generalized across the whole country. Moreover, the majority of previous studies in Ethiopia used fever or diarrhea alone as outcome variables; however, in this study, we were able to see the effect of socio-economic and other demographic predictors on three common childhood health problems and on mothers’ health seeking behavior.

## Conclusion

This study revealed that the number of respondents who sought medical treatment for common childhood illnesses was not large enough. This study identified the main factors related to the inequalities in health care seeking behavior for common childhood illnesses. Interestingly, wealth status, maternal education, paternal education, female workload, and religion were the main contributing factors to the inequalities.

Child morbidity and mortality rates are the main indicators of the efficiency and effectiveness of health care systems. Policy interventions aimed at improving appropriate medical treatment seeking behavior for common childhood illnesses are desirable. Considering this finding, more practical economic policies aimed at moving those in the lower wealth quintile (poorest and poorer) need to be implemented to bridge the gap between the rich and the poor. This would enable more mothers to seek and utilize medical treatments and to improve the general health of the population. Moreover, medical treatment seeking behavior can be improved by strengthening the educational system and improving women’s access to education. A mother with a better education may have greater decision-making power in the household and understand the importance of early treatment and prevention resulting in an increased likelihood of her utilizing healthcare services. Access to good education also impacts socioeconomic status which would enhance medical care seeking behavior for childhood illnesses and general health service utilization. Hence, women’s education serves as a positive feedback mechanism for economic development and national productivity as well as increasing the household wealth status of families [17]. We also recommend that further studies should be done by triangulating the quantitative and qualitative methods to further determine the effect of cultural and religious factors on medical care seeking behaviors of mothers.

## Additional file

Additional file 1: Table S1. Number and proportion of subjects by predictor variable and outcome variables*.* (DOCX 25 kb)

## Abbreviations

CSA: Central Statistical Agency
EDHS: Ethiopian Demographic Health Survey
UN: United Nations
USAID: United State Agency for International Development
