# Supplementary material for: Inequalities in health care utilization for common childhood illnesses in Ethiopia: evidence from the 2011 Ethiopian Demographic and Health Survey
Source: Int J Equity Health. 2017 Apr 21;16:67. doi: 10.1186/s12939-017-0561-7 (PMC5399816; doi:10.1186/s12939-017-0561-7)
Supplement: Additional file 1: Table S1. — Number and proportion of subjects by predictor variable and outcome variables. (DOCX 25 kb) [file 12939_2017_561_MOESM1_ESM.docx]

**Additional file: Table S1. Number and proportion of subjects by predictor variable and outcome variables.**

| Predictor variables | Diarrhea treatment  (N= 1,620) Yes = 576(35%) | | | Cough treatment  (N = 2,134) Yes = 555(26%) | | | Fever treatment  ( N = 2,082) Yes= 604(29%) | | |
| --- | --- | --- | --- | --- | --- | --- | --- | --- | --- |
|  | **Yes**  **N (%)** | **No**  **N (%)** | **Total**  **N (%)** | **Yes**  **N (%)** | **No**  **N (%)** | **Total**  **N (%)** | **Yes**  **N (%)** | **No**  **N (%)** | **Total**  **N (%)** |
| Household wealth index | | | | | | | | | |
| Poorest | 142(26) | 397(74) | 539(33.3) | 127(18.5) | 560(81.5) | 687(32.2) | 151(21) | 565(79) | 716(34.5) |
| Poorer | 83(30) | 191(70) | 274(17) | 77(21.6) | 279(78.4) | 356(16.7) | 88(25) | 268(75) | 356(17.1) |
| Middle | 106(38.8) | 167(61.2) | 273(17) | 91(23.9) | 290(76.1) | 381(17.9) | 94(27) | 250(73) | 344(16.5) |
| Richer | 123(40.5) | 181(59.5) | 304(19) | 103(28.5) | 259(71.5) | 362(17) | 111(34) | 218(66) | 329(15.8) |
| Richest | 122(53) | 108(47) | 230(14) | 156(44.8) | 192(55.2) | 348(16.3) | 174(52) | 163(48) | 337(16.2) |
| Maternal highest education | | | | | | | | | |
| No education | 358(32) | 767(68) | 1125(69) | 322(22.2) | 1,130(77.8) | 1451(68) | 360(25) | 1,065(75) | 1425(68.4) |
| Primary education | 176(41) | 257(59) | 433(27) | 187(31.9) | 399(68.1) | 586(27.5) | 201(36) | 356(64) | 557(26.8) |
| Secondary education | 29(63) | 17(37) | 46(3) | 30(42) | 40(57) | 70(3.3) | 39(53) | 34(47) | 73(4) |
| Higher education | 13(81) | 3(19) | 16(0.99) | 15(57.7) | 11(42.3) | 26(1.22) | 18(66.67) | 9(33.33) | 27(1.3) |
| Husband's highest education | | | | | | | | | |
| No education | 256(31) | 579(69) | 835(51.5) | 237(21.3) | 874(78.7) | 1111(52.1) | 254(23.6) | 815(76.4) | 1069(51.4) |
| Primary education | 248(39) | 395(61) | 643(40) | 229(28) | 590(72) | 819(38.4) | 2545(32) | 546(68.3) | 800(38.3) |
| Secondary education | 54(51) | 51(49) | 105(6.5) | 62(42.5) | 84957.5) | 146(6.8) | 72(51) | 69(49) | 141(7) |
| Higher education | 18(49) | 19(51) | 37(2.3) | 26(44.8) | 32(55.2) | 58(2.7) | 38(53) | 34(47) | 72(3.5) |
| Household religion | | | | | | | | | |
| Orthodox | 166(35) | 303(65) | 469(29) | 179(22.7) | 608(77.3) | 787(37) | 176(26.5) | 487(73.5) | 663(32) |
| Protestant | 157(38) | 257(62) | 414(25.6) | 133(30.9) | 298(69.1) | 431(20.2) | 171(34) | 337(66) | 508(24.4) |
| Muslim | 244(35) | 453(65) | 697(43) | 237(26.7) | 651(73.3) | 888(41.6) | 265(30) | 610(70) | 875(42) |
| Other | 9(22.5) | 31(77.5) | 40(2.5) | 5(17.9) | 23(82.1) | 28(1.3) | 6(17) | 30(83) | 36(2) |
| Maternal age | | | | | | | | | |
| 15-19 | 32(38) | 52(62) | 84(5.2) | 33(33.7) | 65(66.3) | 98(4.6) | 37(34) | 72(66) | 109(6) |
| 20-24 | 130(40.4) | 192(59.6) | 322(20) | 124(28.8) | 307(71.2) | 431(20.2) | 130(34) | 253(66) | 383(18) |
| 25-29 | 196(38) | 318(62) | 514(31.7) | 183(27.4) | 484(72.6) | 667(31.3) | 204(31.4) | 445(68.6) | 649(31.2) |
| 30-34 | 133(39) | 208(61) | 341(21) | 118(28.4) | 297(71.6) | 415(19.5) | 123(30) | 291(70) | 414(20) |
| 35-39 | 48(22) | 172(78) | 220(13) | 55(16.9) | 271(83.1) | 326(15.3) | 68(21) | 249(79) | 317(15) |
| 40-44 | 32(30) | 75(70) | 107(7) | 34(22.5) | 117(77.5) | 151(7.1) | 46(30) | 109(70) | 155(7) |
| 45-49 | 5(16) | 27(84) | 32(2) | 7(15.2) | 39(84.8) | 46(2.2) | 10(18) | 45(82) | 55(3) |
| Household head Sex | | | | | | | | | |
| Male | 467(35) | 855(65) | 1,322(82) | 438(25.2) | 1,298(74.8) | 1736(81.4) | 482(29) | 1,189(71) | 1671(80.3) |
| Female | 109(37) | 189(63) | 298(18) | 116(29.2) | 282(70.8) | 398(18.6) | 136(33) | 275(67) | 411(19.7) |
| Number of household member’s | | | | | | | | | |
| Less than 6 | 271(39) | 431(61) | 702(43) | 282(29.8) | 663(70.2) | 945(44.3) | 304(34) | 600(66) | 904(43.4) |
| 6 and above | 305(33) | 613(67) | 918(57) | 272(22.9) | 917(77.1) | 1189(55.7) | 314(27) | 864(73) | 1178(56.6) |
| Number of unde-5 children | | | | | | | | | |
| Less than 3 | 486(36.5) | 840(63.5) | 1,326(81.9) | 464(26.4) | 1,294(73.6) | 1758(82.4) | 527(30) | 1,210(70) | 1737(83.4) |
| 3 & above | 90(30) | 204(70) | 294(18.2) | 90(23.9) | 286(76.1) | 376(17.6) | 91(26) | 254(74) | 345(16.6) |
| Child birth-order | | | | | | | | | |
| First | 124(46) | 144(54) | 268(16.5) | 127(32.5) | 264(67.5) | 391(18.3) | 150(41) | 217(59) | 367(17.6) |
| Second | 110(40) | 168(60) | 278(17.2) | 108(27.9) | 279(72.1) | 387(18.1) | 109(32) | 231(68) | 340(16.3) |
| Third | 81(34) | 158(66) | 239(14.8) | 92(30.5) | 210(69.5) | 302(14.2) | 101(31) | 226(69) | 327(15.7) |
| Fourth & above | 261(31) | 574(69) | 835(51.5) | 227(21.5) | 827(78.5) | 1054(49.4) | 258(25) | 790(75) | 1048(50.3) |
| Sex of Child | | | | | | | | | |
| Male | 299(35) | 560(65) | 859(53) | 280(26.4) | 781(73.6) | 1061(49.7) | 336(31) | 750(69) | 1086(52) |
| Female | 277(36) | 484(64) | 761(47) | 274(25.5) | 799(74.5) | 1073(50.3) | 282(28) | 714(72) | 996(48) |
| Wanted last child | | | | | | | | | |
| Wanted no more | 47(30) | 111(70) | 158(10) | 40(19.5) | 165(80.5) | 205(9.6) | 47(24) | 153(76) | 200(9.4) |
| Wanted then | 415(36) | 734(64) | 1,149(71) | 408(27) | 1,103(73) | 1511(70.8) | 455(30) | 1,060(70) | 1515(73) |
| Wanted later | 114(36) | 199(64) | 313(19) | 106(25.4) | 312(74.6) | 418(19.6) | 116(32) | 251(68) | 367(17.6) |
| Maternal marital status | | | | | | | | | |
| Married | 507(36) | 914(64) | 1,421(88) | 79(30.3) | 182(69.7) | 261(12.2) | 533(30) | 1,273(70) | 1806(86.7) |
| Otherwise | 69(35) | 130(65) | 199(12) | 475(25.4) | 1,398(74.6) | 1873(87.8) | 85(31) | 191(69) | 276(13.3) |
| Household has radio | | | | | | | | | |
| Yes | 230(40) | 349(60) | 579(36) | 238(30.6) | 540(69.4) | 778(36.5) | 272(35) | 498(65) | 770(37) |
| No | 346(33) | 695(67) | 1,041(64) | 316(23.3) | 1,040(76.7) | 1356(63.5) | 346(26) | 966(74) | 1312(63) |
| Household has TV | | | | | | | | | |
| Yes | 79(50) | 78(50) | 157(10) | 104(48.2) | 112(51.8) | 216(10.1) | 116(52) | 106(48) | 222(11) |
| No | 497(34) | 966(66) | 1,463(90) | 450(23.5) | 1,468(76.5) | 1918(89.9) | 502(27) | 1,358(73) | 1860(89) |
| Concern on lack of provider | | | | | | | | | |
| Big problem | 401(35.5) | 730(65.5) | 1,131(69.8) | 366(26.1) | 1,035(73.9) | 1401(65.7) | 420(30) | 980(70) | 1400(67) |
| Not big problem | 175(31) | 314(69) | 489(30.2) | 188(25.6) | 545(74.4) | 733(34.4) | 198(29) | 484(71) | 682(33) |
| Females workload inside and outside | | | | | | | | | |
| Big problem | 383(33) | 775 (67) | 1,158(71.5) | 359(25.3) | 1,058(74.7) | 1417(66.4) | 229(35) | 424(65) | 653(31) |
| Not a big problem | 193(42) | 269(58) | 462(28.5) | 195(27.2) | 522(72.8) | 717(33.6) | 389(27) | 1,040(73) | 1429(69) |
| Place of residence | | | | | | | | | |
| Urban | 123(54) | 105(46) | 228(14.1) | 136(42.6) | 183(57.4) | 319(15) | 1629 (51) | 159(49) | 321(15) |
| Rural | 453(32.5) | 939(67.5) | 1,392(85.9) | 418(23.1) | 1,397(76.9) | 1815(85) | 456(26) | 1,305(74) | 1761(85) |
